# Sex differences in determinants of COVID-19 severe outcomes – findings from the National COVID Cohort Collaborative (N3C)

**DOI:** 10.1186/s12879-022-07776-7

**Published:** 2022-10-12

**Authors:** Yilin Yoshida, San Chu, Sarah Fox, Yuanhao Zu, Dragana Lovre, Joshua L. Denson, Lucio Miele, Franck Mauvais-Jarvis

**Affiliations:** 1 Section of Endocrinology and Metabolism, Deming Department of Medicine, Tulane University School of Medicine, 1430 Tulane Ave. New Orleans, 70112 New Orleans, LA USA; 2 Pennington Biomedical Research Center, Louisiana State University, Baton Rouge, LA USA; 3 School of Science and Engineering, Tulane University, New Orleans, LA USA; 4 Department of Biostatistics and Data Science, Tulane University School of Public Health and Tropical Medicine, New Orleans, LA USA; 5 Pulmonary and Critical Care, Deming Department of Medicine, Tulane University School of Medicine, New Orleans, LA USA; 6 Department of Genetics, Louisiana State University Health Sciences Center, New Orleans, LA USA

**Keywords:** COVID-19 severity, Sex differences, Comorbidities, Biomarkers

## Abstract

**Objective:**

The impact of comorbidities and biomarkers on COVID-19 severity vary by sex but have not yet been verified in population-based studies. We examined the association of comorbidities, inflammatory biomarkers, and severe outcomes in men and women hospitalized for COVID-19.

**Design:**

This is a retrospective cohort analysis based on the National COVID Cohort Collaborative (N3C). We included 574,391 adult patients admitted for COVID-19 at hospitals or emergency rooms between 01/01/2020 and 12/31/2021.

**Methods:**

We defined comorbidities at or before the first admission for COVID-19 by Charlson Comorbidity Index (CCI) and CCI components. We used the averaged lab values taken within 15 days before or after the admission date to measure biomarkers including c-reactive protein (CRP), ferritin, procalcitonin, N-terminal pro b-type natriuretic peptide (NT proBNP), d-dimer, absolute lymphocyte counts, absolute neutrophil counts, and platelets. Our primary outcome was all-cause mortality; secondary outcomes were invasive mechanical ventilation (IMV) and hospital length of stay (LOS). We used logistic regression adjusted for age, race, ethnicity, visit type, and medications to assess the association of comorbidities, biomarkers, and mortality disaggregating by sex.

**Results:**

Moderate to severe liver disease, renal disease, metastatic solid tumor, and myocardial infarction were the top four fatal comorbidities among patients who were hospitalized for COVID-19 (adjusted odds ratio [aOR] > 2). These four comorbid conditions remained the most lethal in both sexes, with a higher magnitude of risk in women than in men (p-interaction < 0.05). Abnormal elevations of CRP, ferritin, procalcitonin, NT proBNP, neutrophil, and platelet counts, and lymphocytopenia were significantly associated with the risk of death, with procalcitonin and NT proBNP as the strongest predictors (aOR > 2). The association between the abnormal biomarkers and death was stronger in women than in men (p-interaction < 0.05).

**Conclusion:**

There are sex differences in inpatient mortality associated with comorbidities and biomarkers. The significant impact of these clinical determinants in women with COVID-19 may be underappreciated as previous studies stressed the increased death rate in male patients that is related to comorbidities or inflammation. Our study highlights the importance and the need for sex-disaggregated research to understand the risk factors of poor outcomes and health disparities in COVID-19.

**Supplementary Information:**

The online version contains supplementary material available at 10.1186/s12879-022-07776-7.

## Background

Susceptibility to severe coronavirus disease 2019 (COVID-19) is strongly associated with comorbidities and systemic inflammation. However, sex-disaggregated data related to the impact of comorbidities and inflammation in COVID-19 is lacking, and the need to identify how sex may be contributing to these clinical determinants of health remains high [1–6]. Cumulative evidence shows a male predominance in COVID-19 severe outcomes, including increased use of invasive mechanical ventilation (IMV), prolonged hospital stay, and increased mortality [7, 8] that may be attributable to their more prevalent comorbid conditions. However, which specific comorbidities predict higher COVID-19 severity remains unclear. Some evidence suggests that men with macrovascular complications are more susceptible to severe COVID-19 [9–11], whereas, in women, autoimmune disease, dementia, chronic kidney disease, and obesity have been associated with in-hospital death [9, 12].

Additionally, despite the increasing number of studies showing the predictability of inflammatory, coagulation, and cardiac biomarkers with severe COVID-19 outcomes [6], evidence regarding sex differences for these biomarkers and COVID-19 outcomes are limited. While some suggest elevated levels of c-reactive protein (CRP), d-dimer, and thrombocytopenia increase the risk of death among male patients [10], others have shown that inflammatory biomarkers may be more reliable in predicting death in women [13]. Moreover, ferritin and lymphopenia, both key mediators of immune dysregulation via direct immune-suppressive and pro-inflammatory effects, appear to be risk factors specifically for women with COVID-19 [10, 13].

Understanding the degree to which comorbidities and biomarkers affect male and female patients differently has important implications for precision medicine. To this end, we leveraged the National COVID Cohort Collaborative (N3C)[14] to examine sex differences in biological determinants of COVID-19 severe outcomes.

## Methods

### Design and data source

This is a retrospective cohort study using data abstracted from the N3C. N3C is a National Institutes of Health (NIH)-sponsored clinical patient registry for COVID-19. N3C is a centralized repository of electronic health record (EHR) data representing the largest multicenter cohort of COVID-19 cases and controls in the U.S. [14]. As of March 17, 2022, 4,511,826 COVID-19 cases from 71 contributing sites were available on the N3C platform.

### Sample

Our study using N3C data version 3.0 updated on Feburary 4, 2022 included 574,391 adult patients admitted for COVID-19 at hospitals or emergency rooms (ER) from January 1, 2020, to December 31, 2021, with necessary information on demographics. Historical patient data dating back to January 1, 2018, were included to document comorbidities within each health system for all individuals. To identify COVID-19 patients, we first followed the N3C phenotyping guidelines on a basis of a COVID-19–positive polymerase chain reaction or antigen test, or an International Classification of Diseases (ICD)-10-CM diagnostic code for COVID-19 during the same single index encounter [15]. We then restricted our sample to inpatient cases that were defined by the first admission to a hospital or an ER after a COVID-19 positive test or diagnosis.

### Measures

Our primary exposures were comorbidities at or before the admission date. We define comorbidities by Charlson Comorbidity Index (CCI) and CCI components, including myocardial infarction (MI), congestive heart failure, peripheral vascular disease, cerebrovascular disease, dementia, chronic pulmonary disease, rheumatic disease, peptic ulcer disease, liver disease, diabetes, hemiplegia or paraplegia, renal disease, malignancy, metastatic solid tumor, and AIDS/HIV. We used the averaged lab values taken within 15 days prior to or after the admission date to measure biomarkers, including CRP, ferritin, procalcitonin, N-terminal pro b-type natriuretic peptide (NT proBNP), d-dimers, absolute lymphocyte counts, absolute neutrophil counts, and platelets. Cut-offs for biomarkers were set according to previous publications [13, 16]. Most patients had lab values within 15 days after hospital admission. We also chose 15 days before the admission date to identify lab variables because some patients had tests administered at ER before hospitalization (i.e., those who had shown symptoms within the 15 days window after the exposure and sought care in ER before hospitalization). Our primary outcome was all-cause hospital mortality and secondary outcomes were IMV and hospital length of stay (LOS) (≤ 7 or > 7 days). Our covariates included age group (20–39, 40–59, or 60 + years), sex (women or men), race (non-Hispanic white, non-Hispanic black, or others), ethnicity (Hispanics or non-Hispanics), visit type (hospitalization, intensive care, or ER), medications for comorbidities such as anti-hypertensive, lipid-lowering, or anti-diabetic medications. Comorbidities and biomarkers were extracted by code sets (see supplemental Table 1) which are collections of concept sets created and validated by informatician and clinical subject-matter experts. Concepts sets contain standardized terminology corresponding to clinical domains (e.g., LOINC, ICD-10, RxNorm) [17].

**Analysis**: We used the Chi-square test to compare frequencies of comorbidities stratified by sex. We then applied logistic regression for the association between each comorbidity (i.e., CCI component) or the number of comorbidities (i.e., CCI ≥ 2, CCI ≥ 3, CCI ≥ 4, and CCI ≥ 5) and outcomes (all-cause mortality, IMV, LOS) in the overall sample followed by a sex-stratified analysis. A trend analysis was conducted for the increased number of comorbidities for the overall and sex-specific analysis. We used Kruskal-Wallis test to compare medians of continuous biomarker values by sex and used logistic regression to compare the risk of death, IMV, and ≥ 7 days LOS associated with above or below thresholds of biomarkers in the overall sample followed by a sex-stratified analysis. In the multivariable model, we adjusted for age, race, ethnicity, visit type, and any medication use. For biomarker analysis, we additionally adjusted for CCI. We also performed two sensitivity analyses: (1) excluding ER cases to examine the effect of comorbidities and biomarkers in individuals admitted to hospitals only, (2) using a subsample with complete vital information (blood pressure, heart rate, and respiratory rate) to further account for overall health condition at admission. All model-based results were presented with 95% confidence intervals. We used R on the Palantir platform within the N3C Data Enclave for the analysis.

All methods were carried out in accordance with relevant guidelines and regulations. Informed consent was obtained from patients by the original data suppliers (contributing sites) of the NIH N3C. This analysis only involved secondary, de-identified data. The IRB determined that the activities are not human subjects research as defined by the Common Federal Rule. As such, IRB review and approval are not required (Tulane Universit Biomedical IRB, REF# 2021-079).

## Results

A total of 574,391 COVID-19 inpatients were included in the final analysis. The mean age for the cohort was 52-years-old with male patients slightly older than females (54 vs. 51-years-old) (Table 1). Non-Hispanic (NH) Whites accounted for a large portion of the cohort (70%), followed by 25% NH Blacks. Less than 10% of participants were Hispanics. Over 60% of included patients were admitted to ERs and around 38% were admitted to hospitals. More women were admitted to ERs, while more men were admitted to hospitals. Women had higher rates of anti-diabetic medication, lipid-lowering medication, and anti-hypertensive medication use compared to men (9.9% vs. 6.9%, 11.8% vs. 7.8%, and 19.9% vs. 15.2%, respectively). Men had a higher CCI score than women (4.3 vs. 3.7) (Table 1). Men also had a higher percentage of CCI over 3. Chronic pulmonary disease (36%), diabetes without complications (31%), peripheral vascular disease (28%), cerebrovascular disease (27%), mild liver disease (23%), renal disease (21%), and diabetes with complications (21%) were the most prevalent comorbidities in this cohort (Table 1). All comorbidities except peptic ulcer disease showed significant sex differences. Among these the top prevalent conditions (prevalence rate > 20%), women were more likely to have peripheral vascular disease, chronic pulmonary disease, while men were more likely to have renal disease, cerebrovascular disease, or diabetes. Of note, women had a significantly higher proportion of moderate to severe liver disease. Men had higher percentages of abnormalities across all biomarkers compared to women (Table 1).

**Table 1:** Patient characteristics by sex

	All (N = 574,391)	Women (n = 306,638)	Men (n = 267,753)	P-value^1^	X^2^ (DF)
**Age (mean, SD)**	52.3, 18.5	50.7, 18.9	54.1, 17.9	< 0.0001	
**Age group (n, %)**				< 0.0001	6171.1 (2)
20–39	167,186, 29.1%	102,537, 33.4%	64,649, 24.1%		
40–59	183,707, 32.0%	94,247, 30.7%	89,460, 33.4%		
60 or 60+	223,498, 38.9%	109,854, 35.8%	113,644, 42.4%		
**Race (n,%)**				< 0.0001	1317.5 (3)
Non-Hispanic (NH) White	399,690, 69.6%	208,433, 68.0%	191,257, 71.4%		
NH Black	140,712, 24.5%	80,956, 26.4%	59,756, 22.3%		
Others	33,989, 5.9%	17,249, 5.6%	16,740, 6.3%		
**Ethnicity (n,%)**				0.4335	0.6134 (1)
Hispanics	53,610, 9.3%	28,533, 9.3%	25,077, 9.4%		
Non-Hispanics	520,781, 90.7%	278,105, 90.7%	242,676, 90.6%		
**Visit Type**				< 0.0001	1370.1 (2)
Hospital	215,661, 37.6%	108,516, 35.4%	107,145, 40.3%		
ER	357,686, 62.3%	197,675, 64.5%	160,011, 59.8%		
Intensive care	1044, 0.1%	447, 0.1%	597, 0.2%		
**Medication at admission (n, %)**					
Anti-diabetic	47,752, 8.3%	21,296, 6.9%	26,456, 9.9%	< 0.0001	1615.8 (1)
Lipid-lowering	55,617, 9.7%	23,923, 7.8%	31,694, 11.8%	< 0.0001	2661.1 (1)
Anti-hypertensive	99,877, 17.4%	46,648, 15.2%	53,229, 19.9%	< 0.0001	2167.2 (1)
Unknown	449,075, 78.2%	248,376, 81.0%	200,699, 75.0%	< 0.0001	3060.2 (1)
**Vitals at admission** ^**2**^					
SBP (mean, SD)	129.7, 19.8	128.3, 19.3	131.2, 20.1	< 0.0001	
Heart rates (mean, SD)	87.6, 15.7	88.1, 15.5	87.0, 15.9	< 0.0001	
Respiratory rate (mean, SD)	19.7, 23.1	19.4, 21.7	20.1, 24.7	< 0.0001	
**Charlson Comorbidity Index (CCI)**					
CCI (mean, SD)	4.0, 4.8	3.7, 4.6	4.3, 5.0	< 0.0001	
CCI ≥ 3 (n, %)	259,545, 45.2%	131,380, 42.8%	128,165, 47.9%	< 0.0001	1455.0 (1)
**CCI components**					
Myocardial infarction (n, %)	45,870, 8%	18,596, 6.1%	27,274, 10.2%	< 0.0001	3304.2 (1)
Congestive heart failure (n, %)	75,028, 13.1%	35,193, 11.5%	39,835, 14.9%	< 0.0001	1455.2 (1)
Peripheral vascular disease (n, %)	164,612, 28.7%	89,831, 29.3%	74,781, 27.9%	< 0.0001	130.5 (1)
Cerebrovascular disease (n, %)	153,030, 26.6%	79,494, 25.9%	73,536, 27.5%	< 0.0001	173.3 (1)
Dementia (n, %)	28,619, 5.0%	14,923, 4.9%	13,696, 5.1%	< 0.0001	18.6 (1)
Chronic pulmonary disease (n, %)	206,042, 35.9%	113,326, 37.0%	92,716, 34.6%	< 0.0001	337.3 (1)
Rheumatic disease (n, %)	115,071, 20.0%	66,075, 21.5%	48,996, 18.3%	< 0.0001	941.8 (1)
Peptic ulcer disease (n, %)	23,053, 4.0%	12,338, 4.0%	10,715, 4.0%	0.6793	0.171 (1)
Mild liver disease (n, %)	132,564, 23.1%	64,551, 21.1%	68,013, 25.4%	< 0.0001	1523.5 (1)
Diabetes without chronic complication (n, %)	177,558, 30.9%	89,652, 29.2%	87,906, 32.8%	< 0.0001	864.3 (1)
Diabetes with chronic complication (n, %)	120,697, 21.0%	57,428, 18.7%	63,269, 23.6%	< 0.0001	2068.6 (1)
Hemiplegia or paraplegia (n, %)	20,399, 3.6%	9843, 3.2%	10,556, 3.9%	< 0.0001	223.7 (1)
Renal disease (n, %) %	131,973, 23.0%	59,184, 19.3%	72,789, 27.2%	< 0.0001	5020.3 (1)
Any malignancy, including lymphoma and leukemia, except malignant neoplasm of skin (n, %)	94,283, 16.4%	49,480, 16.1%	44,803, 16.7%	< 0.0001	37.0 (1)
Moderate or severe liver disease (n, %)	100,351, 17.5%	43,669, 14.2%	56,682, 21.2%	< 0.0001	4758.2 (1)
Metastatic solid tumor (n, %)	17,000, 3.0%	8534, 2.8%	8466, 3.2%	< 0.0001	71.3 (1)
AIDS/HIV (n, %)	3821, 0.7%	1213, 0.4%	2608, 1.0%	< 0.0001	722.9 (1)
**Biomarkers**					
C-reactive protein (median IQR)	17 (6, 69.3)	15 (5, 60.5)	19.1 (6.9, 77.3)	< 0.0001	
C-reaction > 30 mg/L (n, %)	71,721, 39.1%	31,394, 36.5%	40,329, 41.4%	< 0.0001	886.3 (1)
Ferritin (median IQR)	462 (196, 985)	313 (132.6, 666)	643 (299.5, 1280)	< 0.0001	
Ferritin > 300 ng/mL (n, %)	106,127, 57.4%	40,379, 51.3%	65,748, 62.0%	< 0.0001	10,019 (1)
Procalcitonin (median IQR)	0.17 (0.09, 0.54)	0.14 (0.07, 0.48)	0.2 (0.1, 0.6)	< 0.0001	
Procalcitonin >0.1 ng/mL (n, %)	61,840, 72.0%	24,565, 65.9%	37,275, 76.6%	< 0.0001	218.2 (1)
NT proBNP pg/mL (median IQR)	240 (65, 1090)	223 (62, 998)	258 (68, 1162)	< 0.0001	
NT proBNP > 400 pg/mL (n, %)	29,568, 40.5%	13,400, 39.1%	16,168, 41.8%	< 0.0001	56.9 (1)
D-dimers (median IQR) ng/mL	178 (1, 631)	172 (1, 601)	184 (1, 665)	< 0.0001	
D-dimers > 250 ng/mL (n, %)	70,208, 44.0%	33,920, 43.3%	36,288, 44.6%	< 0.0001	26.5 (1)
Absolute lymphocyte count 10^3^/uL (median IQR)	1.05 (0.8, 1.7)	1.15 (0.9, 1.84)	1 (0.7, 1.5)	< 0.0001	
Absolute lymphocyte count < 1*10^3^ (n, %)	115,281, 34.6%	50,577, 29.1%	64,704, 40.7%	< 0.0001	4941.4 (1)
Absolute neutrophil count 10^3^/uL (median IQR)	4.44 (3, 6.9)	4.2 (3, 6.5)	4.7 (3.05, 7.1)	< 0.0001	
Absolute neutrophil count > 1.8*103/uL (n, %)	349,838, 94.1%	178,929, 93.1%	170,909, 95.2%	< 0.0001	732.8 (1)
Platelet count 10^3^/uL (median IQR)	210 (164, 267.5)	222 (175, 280)	196 (153, 251)	< 0.0001	
Platelet count < 150*10^3^/uL (n %)	74,715, 18.0%	29,619, 13.7%	45,096, 22.9%	< 0.0001	5891.1 (1)

^1^ p-value is from chi-squire test for frequencies (all categorical variables) or Kruskal-Wallis test for medians (continuous biomarkers); ^2^ n for SBP measurements 159,867, n for heart rate measurements 224,192, and n for respiratory rate measurement 193,373; ^3^ n for c-reactive proteins 183,386, n for ferritin 184,836, n for procalcitonin 85,916, n for NT proBNP 72,961, n for d-dimer 159,566, n for absolute lymphocyte count 333,255, n for absolute neutrophil count 371,884, n for platelet count 413,970

## Comorbidities

### Primary outcome - mortality

**Fig. 1:**
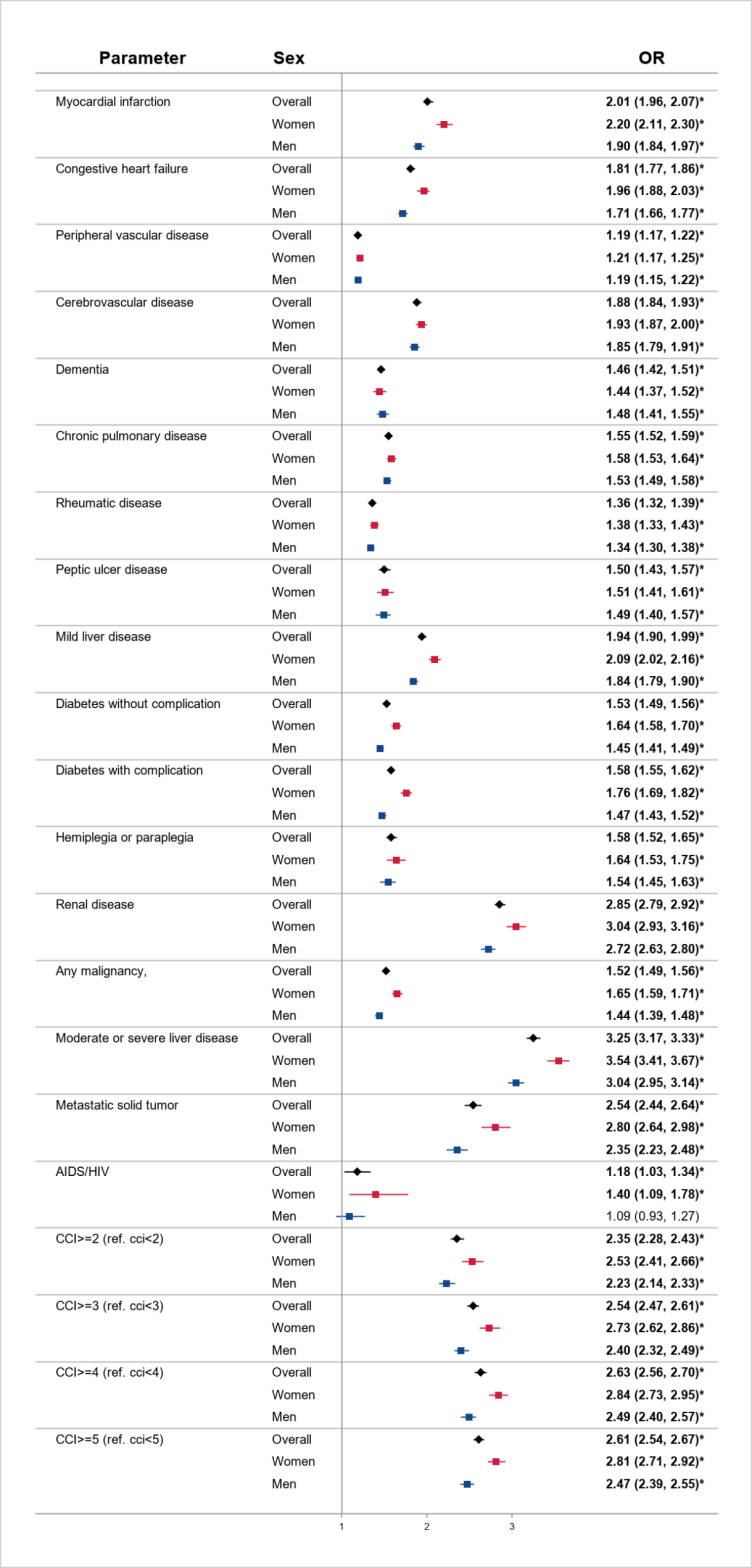
The Association between Comorbidities and Mortality in Men and Women with COVID-19

All individual CCI components were significantly associated with an elevated risk of death in COVID-19 patients (Fig. 1). Notably, in the adjusted model, moderate to severe liver disease, renal disease, metastatic solid tumor, and MI were the top four fatal comorbidities among patients who were hospitalized for COVID-19 (all associated with over 2-fold higher risk of death) (Fig. 1). These four comorbid conditions remained the most lethal in both female and male patients with COVID-19, with the magnitude of risk higher in women than in men (p-interaction < 0.05, not shown in the figure). In addition, the magnitude of association between congestive heart failure, cerebrovascular disease, mild liver disease, diabetes with or without complications, any malignancy, or AIDS/HIV and mortality was higher in women compared to men. The increased number of CCI components was associated with an increased risk of death in men and women (p-trend < 0.05, not shown in the figure) with a stronger incremental effect in women (Fig. 1).

### Secondary outcomes – IMV and LOS

Moderate to severe liver disease, renal disease, and cerebrovascular disease were the top three comorbid conditions associated with the risk of IMV in patients with COVID-19 (ORs > 2). Consistent with findings of sex differences in comorbidities and death, the effect of comorbidities on IMV was greater in women compared to men (Supplemental Fig. 1). The association between comorbidities and hospital LOS was modest. Moderate to severe liver disease was associated with more than 50% odds of LOS over one week. Despite a small effect of comorbidities on LOS, the magnitude of the association was bigger in women than in men (Supplemental Fig. 2).

## Biomarkers

### Primary outcome - mortality

**Fig. 2:**
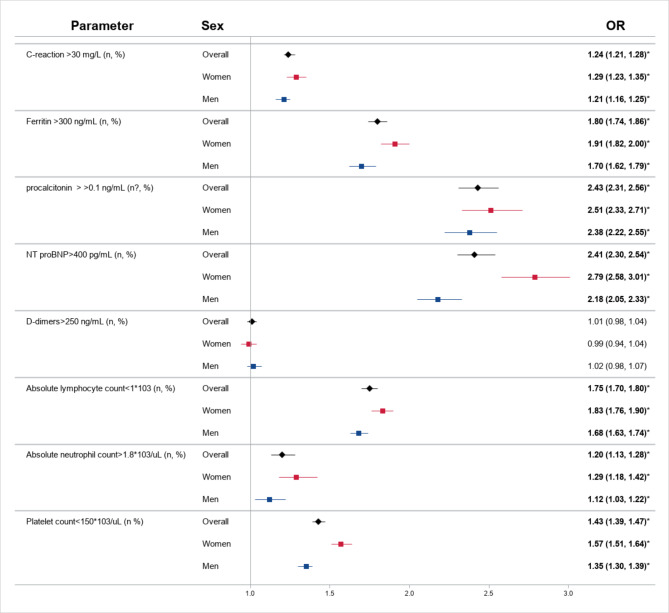
The Association between Biomarkers and Mortality in Men and Women with COVID-19

Seven biomarkers with abnormal levels were significantly associated with COVID-19 mortality, among which NT proBNP > 400 pg/mL was associated with a 2.4-fold higher odds of death, procalcitonin > 100 ng/L, lymphocyte counts < 1 × 10^3^/uL, and ferritin > 300 ng/mL were associated with over 50% higher odds of death (Fig. 2). Above normal thresholds of CRP, neutrophil, and platelet counts were associated with over 20% higher odds of death. Of note, there were significant sex differences (p interaction < 0.05) in the association of CRP, ferritin, NT proBNP, lymphopenia with increased neutrophils, and COVID-19 death, where the abnormal levels of biomarkers showed a more pronounced effect in women than in men (Fig. 2).

### Secondary outcomes – IMV and LOS

Elevated CRP and neutrophil counts were associated with more than 2-fold higher odds of IMV. Elevated NT proBNP, ferritin, d-dimer, platelets, and reduced lymphocytes were all significantly associated with a modest risk of IMV (ORs < 2) (Supplemental Fig. 3). Like the findings for mortality, a marked sex difference was observed for the association between lymphopenia and risk of IMV. CRP, ferritin, NT proBNP, d-dimer, lymphocytes, neutrophils, and platelet counts were associated with LOS over 7 days with modest ORs (< 2) (Supplemental Fig. 4).

We performed two **sensitivity analyses**. By excluding ER cases (n = 216,705), results were similar to that of the main analysis with moderate to severe liver disease, renal disease, and metastatic solid tumor being the leading fatal comorbidities, and elevated procalcitonin, NT proBNP, and ferritin being the top biomarkers related to COVID-19 death (Supplemental Fig. 5). Additionally, sex differences in COVID-19 death were associated with most comorbidities except for dementia, peptic ulcer disease, and HIV/AIDS. Like in the main analysis, the effect of comorbidities was stronger in women relative to men. Sex differences in biomarkers with regard to death were not significant except for NT proNBP and lymphopenia with the effect more evident in female versus male patients (p interaction < 0.05) (Supplemental Fig. 6).

We also replicated the analysis in a subsample with complete vital information at admission (n = 93,967). With additional adjustment of vitals, moderate to severe liver disease, renal disease, and metastatic solid tumor remained the top fatal comorbidities among COVID-19 patients with a bigger magnitude of association observed in women compared to men (Supplemental Fig. 7). Findings for biomarkers predicting death were similar to the main analysis with procalcitonin, NT proBNP, ferritin, and lymphocytes showing more than 50% odds associated with death. We also observed a bigger magnitude of association between ferritin, NT proBNP, and lymphopenia in women than men (Supplemental Fig. 8).

## Discussion

Results from this national cohort showed that moderate to severe liver disease, CKD, metastatic cancer, and MI are the top comorbidities associated with COVID-19 death. These chronic conditions shared similar risk factors for severe COVID-19, such as advancing age, obesity, and diabetes. In addition, they are all associated with immune dysregulation and/or coagulopathy, which contributes to a more severe COVID-19 course [18]. Patients with chronic liver disease and especially cirrhosis, have multiple mechanisms of immune dysfunction that can lead to increased susceptibility to infection and an aberrant inflammatory response during an infection – collectively known as cirrhosis-associated immune dysfunction. This immune dysfunction including reduced components of the complement system, macrophage activation, impaired lymphocyte and neutrophil function, toll-like receptor upregulation, and intestinal dysbiosis [19, 20], may play a role in COVID-19 severity. Patients with CKD have a higher rate of all-type infections than the general population. Similar to liver disease, CKD causes marked alternations in the immune system, including persistent systemic inflammation and acquired immunosuppression, which may explain the higher risk of COVID-19 severe outcomes among individuals with CKD [21]. Likewise, among patients with underlying CVD, especially MI, SARS-CoV-2 may directly damage myocardial cells, trigger systemic inflammatory responses, destabilize coronary plaque, and aggravate hypoxia, therefore, putting patients with CVD at high risk of myocardial injury and death of COVID-19 [22]. Patients with cancer, in general, are also older and have more comorbidities than the general population. In addition, their immune system is frequently compromised as a consequence of anticancer treatments, the disease itself, or both, placing them at increased risk for COVID-19 severity [23].

Among a small number of studies that included sex-disaggregated analysis since the outbreak, findings of the impact of chronic liver disease, CKD, cancer, and CVD on COVID-19 severe outcomes were mixed. The association between CKD and COVID-19 death in this study is consistent with our earlier analysis based on a cohort of patients hospitalized for COVID-19 in New Orleans, US [13]. However, studies in Italy and France have reported that men with CKD had a higher risk of death than women [9, 10]. The evidence before the pandemic suggests women with CKD appeared to have a survival advantage over men, with the difference attenuated when health behaviors and sex hormones were accounted for [24]. With respect to chronic liver disease, although women have a slower progression of fibrosis and decreased incidence of cirrhosis pre-transplantation, after liver transplantation, women have a higher risk of advanced fibrosis and graft loss in HCV-related disease, and a higher risk of death,compared to men. Additionally, women who survived the acute state of MI also had a greater chance of death within one year than men [25]. Sex differences in metastatic cancer and COVID-19 severity have not been well described but could be due to hormones, viral infections, carcinogenic susceptibility, and health care access and utilization [26]. The biological and psychosocial factors explaining the sex differences in CKD, liver disease, cancer, and CVD and COVD-19 death deserve further investigation.

Evidence suggests that a high degree of systemic inflammation mediated by the cytokine release syndrome in hospitalized COVID-19 patients is responsible for COVID-19 severity [27]. Consistently, our study demonstrated that both men and women presented increased inflammation and coagulation, as suggested by the higher levels of CRP, ferritin, procalcitonin, NT proBNP and lymphopenia were at a higher risk of death. Our findings also add value to previous studies by demonstrating that the effect of abnormal levels of the biomarkers is more pronounced in women than in men. Findings on sex differences in inflammatory biomarkers and COVID-19 outcomes appeared to be heterogeneous. As observed in Wuhan [33], men were more likely to exhibit systemic inflammation compared to women, with increased procalcitonin, ferritin, and a greater percentage of monocytes. However, others have reported that ferritin, neutrophil, and procalcitonin were independent predictors of death in women [7]. Our findings from this national cohort indicate the exaggerated innate immune response could be a greater predictor of COVID-19 severity in women than in men and suggest further research to elucidate the possible mechanism underlying these sex disparities in immune, inflammatory, and hypercoagulability markers.

Our findings need to be interpreted in light of their limitations. Due to data unavailability and challenges in parameter mapping, we accounted for limited clinical and psychosocial covariates, therefore, the analysis is subject to residual confounding. However, we have adjusted for key demographic variables, medications, and additionally for vital measurements in a sensitivity analysis. Additionally, the historical patient data in N3C was only dating back to January 1, 2018. We were not able to capture comorbidities and corresponding treatment before that date. Further, we were not able to define sex/gender including a collection of gender-related variables, such as identity, role, or relations. Another limitation of the data source is that despite the continuous effort in data harmonization, N3C includes data aggregated from different health systems with different local practices, regulations, and data models, which may result in reporting differences. Additionally, we focused on major comorbidities defined by CCI. Obesity, a risk factor or precursor of many included comorbidities such as diabetes and CKD was not included. Despite these limitations, this large patient-level COVID-19 data registry that covers heterogeneous populations in the US provided an unparalleled opportunity for robust sex-disaggregated analysis.

## Conclusion

Our study based on the largest COVID-19 cohort in the US identified marked sex differences in inpatient mortality associated with comorbidities and biomarkers. Notably, the associations were more pronounced in women compared to men. The significant impact of clinical determinants in women with COVID-19 may be underappreciated as previous reports stressed the disproportionate death rate in male patients related to their underlying comorbidities and susceptibility to infection and disease severity. Considering the exacerbating effects of the pandemic on health disparities, the public health and clinical communities must strengthen sex-specific services and support to patients, especially those with comorbidities such as moderate to severe liver disease, CKD, metastatic cancer, and MI. Additionally, the differential effect of biomarkers in men and women may aid the development of clinical care management algorisms to improve patients’ outcomes. These laboratory parameters that have shown significant association with COVID-19 mortality in our study may help differentiate the severely ill patients and allow for the appropriate allocation of healthcare resources.

## Electronic supplementary material

Below is the link to the electronic supplementary material.

Supplementary Material 1

## Data Availability

The N3C level 2 data (de-identified data) which was used for this publication are available for researchers from U.S. or non-U.S. insitituions with completion of IT training and DUR approval from the N3C publication committee.

## Abbreviations

CRP: C-reactive protein.
NT proBNP: N-terminal pro b-type natriuretic peptide.
CKD: Chronic kidney disease.
MI: Myocardial infarction.

## References

[1] Bhatraju PK (2020). Covid-19 in Critically Ill Patients in the Seattle Region - Case Series. N Engl J Med.

[2] Grasselli G (2020). Baseline Characteristics and Outcomes of 1591 Patients Infected With SARS-CoV-2 Admitted to ICUs of the Lombardy Region, Italy. JAMA.

[3] Guan W-j (2020). Clinical Characteristics of Coronavirus Disease 2019 in China. N Engl J Med.

[4] Onder G, Rezza G, Brusaferro S (2020). Case-Fatality Rate and Characteristics of Patients Dying in Relation to COVID-19 in Italy. JAMA.

[5] Suleyman G (2020). Clinical Characteristics and Morbidity Associated With Coronavirus Disease 2019 in a Series of Patients in Metropolitan Detroit. JAMA Netw Open.

[6] Malik P (2021). Biomarkers and outcomes of COVID-19 hospitalisations: systematic review and meta-analysis. BMJ Evid Based Med.

[7] Jin J-M, et al., *Gender Differences in Patients With COVID-19: Focus on Severity and Mortality*. Frontiers in Public Health, 2020. 8. 10.3389/fpubh.2020.00152 PMC7201103 32411652

[8] Griffith DM (2020). Men and COVID-19: A Biopsychosocial Approach to Understanding Sex Differences in Mortality and Recommendations for Practice and Policy Interventions. Prev Chronic Dis.

[9] Raparelli V (2020). Sex differences in clinical phenotype and transitions of care among individuals dying of COVID-19 in Italy. Biol Sex Differ.

[10] Tramunt B (2021). Sex disparities in COVID-19 outcomes of inpatients with diabetes: insights from the CORONADO study. Eur J Endocrinol.

[11] Cheng R, et al., *Sex Differences in the Incidence and Risk Factors of Myocardial Injury in COVID-19 Patients: A Retrospective Cohort Study*. Frontiers in Physiology, 2021. 12(110). 10.3389/fphys.2021.632123 PMC7920972 33664674

[12] Naaraayan A (2021). Sex Disparity in the Effect of Obesity in Hospitalized COVID-19 Patients: A Retrospective Cohort Study From the New York City Metropolitan Area. Cureus.

[13] Yoshida Y, Gillet BM, Wilson SA, Ahmed SM, Tirumalasetty SJ, Zu S, Lovre Y, Denson D, Mauvais-Jarvis JL. F, Clinical Characteristics and Outcomes in Women and Men Hospitalized for Coronavirus Disease-2019 in New Orleans. Biol Sex Differ; 2021. 10.1186/s13293-021-00359-2 PMC7863061 33546750

[14] Haendel MA (2021). The National COVID Cohort Collaborative (N3C): Rationale, design, infrastructure, and deployment. J Am Med Inform Assoc.

[15] Bennett TD (2021). Clinical Characterization and Prediction of Clinical Severity of SARS-CoV-2 Infection Among US Adults Using Data From the US National COVID Cohort Collaborative. JAMA Netw Open.

[16] Xie J, et al. Metabolic Syndrome and COVID-19 Mortality Among Adult Black Patients in New Orleans. Diabetes Care; 2020. 10.2337/dc20-1714 PMC7783937 32843337

[17] Anzalone AJ, et al., *Higher hospitalization and mortality rates among SARS-CoV-2 infected Persons in Rural America.* medRxiv, 2021: p. 2021.10.05.21264543. 10.1111/jrh.12689 PMC9349606 35758856

[18] Sinha P, Matthay MA, Calfee CS, *Is a “Cytokine Storm” Relevant to COVID-19?* JAMA Internal Medicine, 2020. **180**(9): p. 1152–1154. 10.1001/jamainternmed.2020.3313 32602883

[19] Albillos A, Lario M, Álvarez-Mon M (2014). Cirrhosis-associated immune dysfunction: distinctive features and clinical relevance. J Hepatol.

[20] Noor MT, Manoria P (2017). Immune Dysfunction in Cirrhosis. J Clin Transl Hepatol.

[21] D’Marco L (2020). Coronavirus disease 2019 in chronic kidney disease. Clin Kidney J.

[22] Guo T (2020). Cardiovascular Implications of Fatal Outcomes of Patients With Coronavirus Disease 2019 (COVID-19). JAMA Cardiol.

[23] Chavez-MacGregor M (2022). Evaluation of COVID-19 Mortality and Adverse Outcomes in US Patients With or Without Cancer. JAMA Oncol.

[24] Ricardo AC (2019). Sex-Related Disparities in CKD Progression. J Am Soc Nephrol.

[25] Bugiardini R, Cenko E (2020). Sex differences in myocardial infarction deaths. The Lancet.

[26] Cook MB (2011). Sex disparities in cancer mortality and survival. Cancer Epidemiol Biomarkers Prev.

[27] Moore JB, June CH (2020). Cytokine release syndrome in severe COVID-19. Science.

