# Supplementary material for: Sex differences in determinants of COVID-19 severe outcomes – findings from the National COVID Cohort Collaborative (N3C)
Source: BMC Infect Dis. 2022 Oct 12;22:784. doi: 10.1186/s12879-022-07776-7 (PMC9555705; doi:10.1186/s12879-022-07776-7)
Supplement: Supplementary file 1 — Supplementary Material 1 [file 12879_2022_7776_MOESM1_ESM.pdf]

Supplemental Fig 1. The Association between Comorbidities and Invasive Mechanical Ventilation in Men and Women with COVID-19

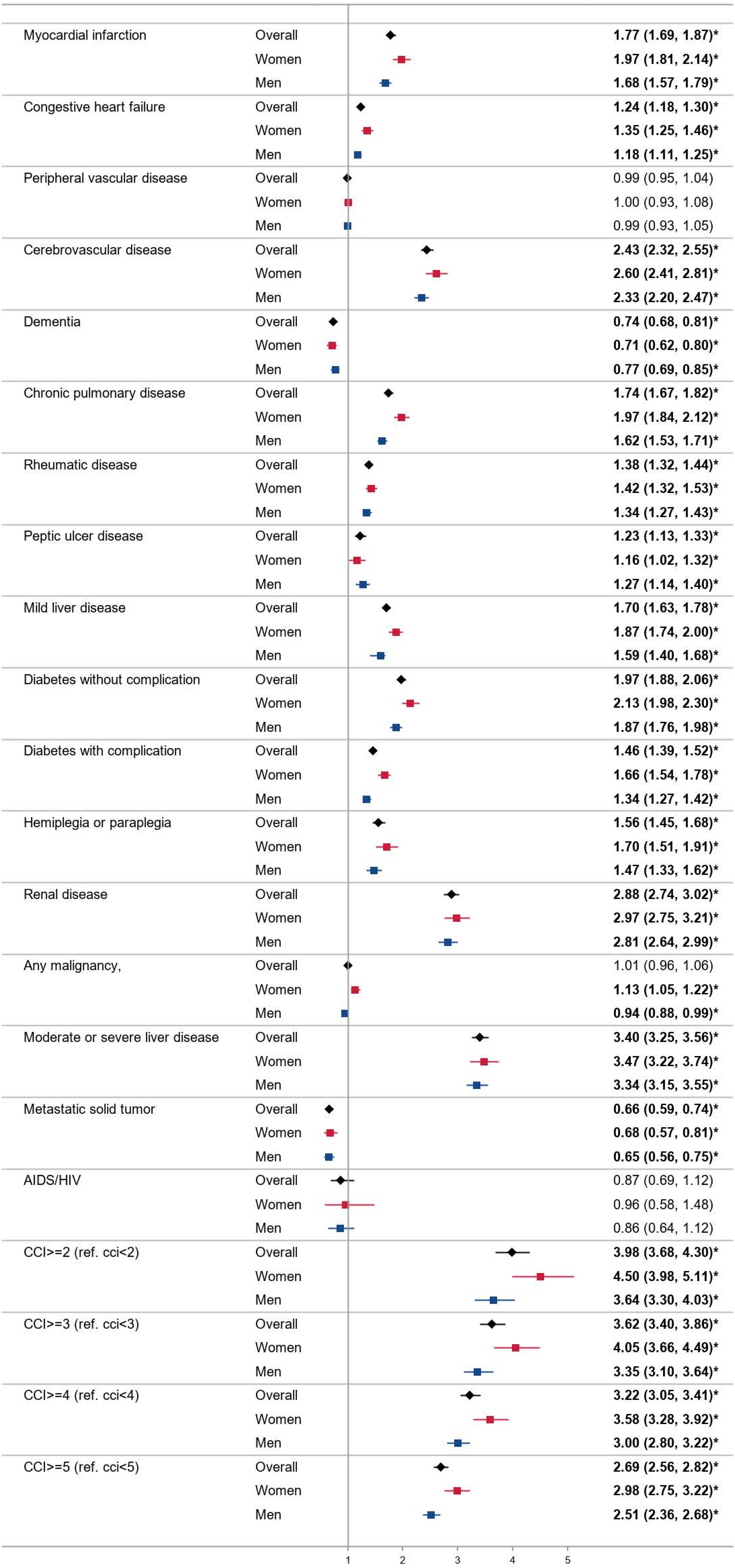

Parameter	Sex	OR
Myocardial infarction	Overall	**1.77 (1.69, 1.87)***
	Women	**1.97 (1.81, 2.14)***
	Men	**1.68 (1.57, 1.79)***
Congestive heart failure	Overall	**1.24 (1.18, 1.30)***
	Women	**1.35 (1.25, 1.46)***
	Men	**1.18 (1.11, 1.25)***
Peripheral vascular disease	Overall	0.99 (0.95, 1.04)
	Women	1.00 (0.93, 1.08)
	Men	0.99 (0.93, 1.05)
Cerebrovascular disease	Overall	**2.43 (2.32, 2.55)***
	Women	**2.60 (2.41, 2.81)***
	Men	**2.33 (2.20, 2.47)***
Dementia	Overall	**0.74 (0.68, 0.81)***
	Women	**0.71 (0.62, 0.80)***
	Men	**0.77 (0.69, 0.85)***
Chronic pulmonary disease	Overall	**1.74 (1.67, 1.82)***
	Women	**1.97 (1.84, 2.12)***
	Men	**1.62 (1.53, 1.71)***
Rheumatic disease	Overall	**1.38 (1.32, 1.44)***
	Women	**1.42 (1.32, 1.53)***
	Men	**1.34 (1.27, 1.43)***
Peptic ulcer disease	Overall	**1.23 (1.13, 1.33)***
	Women	**1.16 (1.02, 1.32)***
	Men	**1.27 (1.14, 1.40)***
Mild liver disease	Overall	**1.70 (1.63, 1.78)***
	Women	**1.87 (1.74, 2.00)***
	Men	**1.59 (1.40, 1.68)***
Diabetes without complication	Overall	**1.97 (1.88, 2.06)***
	Women	**2.13 (1.98, 2.30)***
	Men	**1.87 (1.76, 1.98)***
Diabetes with complication	Overall	**1.46 (1.39, 1.52)***
	Women	**1.66 (1.54, 1.78)***
	Men	**1.34 (1.27, 1.42)***
Hemiplegia or paraplegia	Overall	**1.56 (1.45, 1.68)***
	Women	**1.70 (1.51, 1.91)***
	Men	**1.47 (1.33, 1.62)***
Renal disease	Overall	**2.88 (2.74, 3.02)***
	Women	**2.97 (2.75, 3.21)***
	Men	**2.81 (2.64, 2.99)***
Any malignancy,	Overall	1.01 (0.96, 1.06)
	Women	**1.13 (1.05, 1.22)***
	Men	**0.94 (0.88, 0.99)***
Moderate or severe liver disease	Overall	**3.40 (3.25, 3.56)***
	Women	**3.47 (3.22, 3.74)***
	Men	**3.34 (3.15, 3.55)***
Metastatic solid tumor	Overall	**0.66 (0.59, 0.74)***
	Women	**0.68 (0.57, 0.81)***
	Men	**0.65 (0.56, 0.75)***
AIDS/HIV	Overall	0.87 (0.69, 1.12)
	Women	0.96 (0.58, 1.48)
	Men	0.86 (0.64, 1.12)
CCI>=2 (ref. cci<2)	Overall	**3.98 (3.68, 4.30)***
	Women	**4.50 (3.98, 5.11)***
	Men	**3.64 (3.30, 4.03)***
CCI>=3 (ref. cci<3)	Overall	**3.62 (3.40, 3.86)***
	Women	**4.05 (3.66, 4.49)***
	Men	**3.35 (3.10, 3.64)***
CCI>=4 (ref. cci<4)	Overall	**3.22 (3.05, 3.41)***
	Women	**3.58 (3.28, 3.92)***
	Men	**3.00 (2.80, 3.22)***
CCI>=5 (ref. cci<5)	Overall	**2.69 (2.56, 2.82)***
	Women	**2.98 (2.75, 3.22)***
	Men	**2.51 (2.36, 2.68)***

Supplemental Fig 2. The Association between Comorbiditied and Hospital Length of Stay > 1 Week in Men and Women with COVID-19

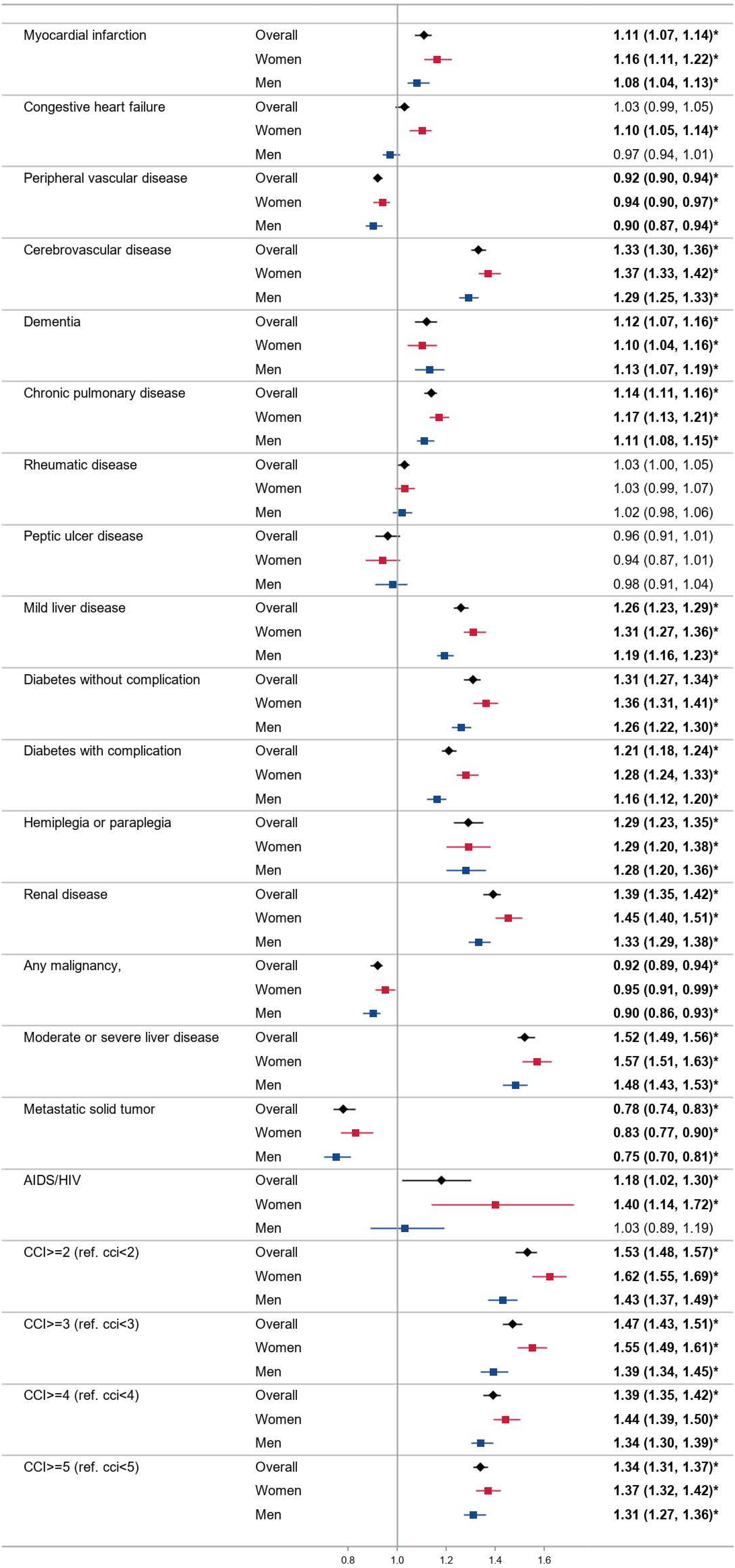

Parameter	Sex	OR
Myocardial infarction	Overall	**1.11 (1.07, 1.14)***
	Women	**1.16 (1.11, 1.22)***
	Men	**1.08 (1.04, 1.13)***
Congestive heart failure	Overall	1.03 (0.99, 1.05)
	Women	**1.10 (1.05, 1.14)***
	Men	0.97 (0.94, 1.01)
Peripheral vascular disease	Overall	**0.92 (0.90, 0.94)***
	Women	**0.94 (0.90, 0.97)***
	Men	**0.90 (0.87, 0.94)***
Cerebrovascular disease	Overall	**1.33 (1.30, 1.36)***
	Women	**1.37 (1.33, 1.42)***
	Men	**1.29 (1.25, 1.33)***
Dementia	Overall	**1.12 (1.07, 1.16)***
	Women	**1.10 (1.04, 1.16)***
	Men	**1.13 (1.07, 1.19)***
Chronic pulmonary disease	Overall	**1.14 (1.11, 1.16)***
	Women	**1.17 (1.13, 1.21)***
	Men	**1.11 (1.08, 1.15)***
Rheumatic disease	Overall	1.03 (1.00, 1.05)
	Women	1.03 (0.99, 1.07)
	Men	1.02 (0.98, 1.06)
Peptic ulcer disease	Overall	0.96 (0.91, 1.01)
	Women	0.94 (0.87, 1.01)
	Men	0.98 (0.91, 1.04)
Mild liver disease	Overall	**1.26 (1.23, 1.29)***
	Women	**1.31 (1.27, 1.36)***
	Men	**1.19 (1.16, 1.23)***
Diabetes without complication	Overall	**1.31 (1.27, 1.34)***
	Women	**1.36 (1.31, 1.41)***
	Men	**1.26 (1.22, 1.30)***
Diabetes with complication	Overall	**1.21 (1.18, 1.24)***
	Women	**1.28 (1.24, 1.33)***
	Men	**1.16 (1.12, 1.20)***
Hemiplegia or paraplegia	Overall	**1.29 (1.23, 1.35)***
	Women	**1.29 (1.20, 1.38)***
	Men	**1.28 (1.20, 1.36)***
Renal disease	Overall	**1.39 (1.35, 1.42)***
	Women	**1.45 (1.40, 1.51)***
	Men	**1.33 (1.29, 1.38)***
Any malignancy,	Overall	**0.92 (0.89, 0.94)***
	Women	**0.95 (0.91, 0.99)***
	Men	**0.90 (0.86, 0.93)***
Moderate or severe liver disease	Overall	**1.52 (1.49, 1.56)***
	Women	**1.57 (1.51, 1.63)***
	Men	**1.48 (1.43, 1.53)***
Metastatic solid tumor	Overall	**0.78 (0.74, 0.83)***
	Women	**0.83 (0.77, 0.90)***
	Men	**0.75 (0.70, 0.81)***
AIDS/HIV	Overall	**1.18 (1.02, 1.30)***
	Women	**1.40 (1.14, 1.72)***
	Men	1.03 (0.89, 1.19)
CCI>=2 (ref. cci<2)	Overall	**1.53 (1.48, 1.57)***
	Women	**1.62 (1.55, 1.69)***
	Men	**1.43 (1.37, 1.49)***
CCI>=3 (ref. cci<3)	Overall	**1.47 (1.43, 1.51)***
	Women	**1.55 (1.49, 1.61)***
	Men	**1.39 (1.34, 1.45)***
CCI>=4 (ref. cci<4)	Overall	**1.39 (1.35, 1.42)***
	Women	**1.44 (1.39, 1.50)***
	Men	**1.34 (1.30, 1.39)***
CCI>=5 (ref. cci<5)	Overall	**1.34 (1.31, 1.37)***
	Women	**1.37 (1.32, 1.42)***
	Men	**1.31 (1.27, 1.36)***

Supplemental Fig 3. The Association between Biomarkers and Invasive Mechanical Ventilation in Men and Women with COVID-19

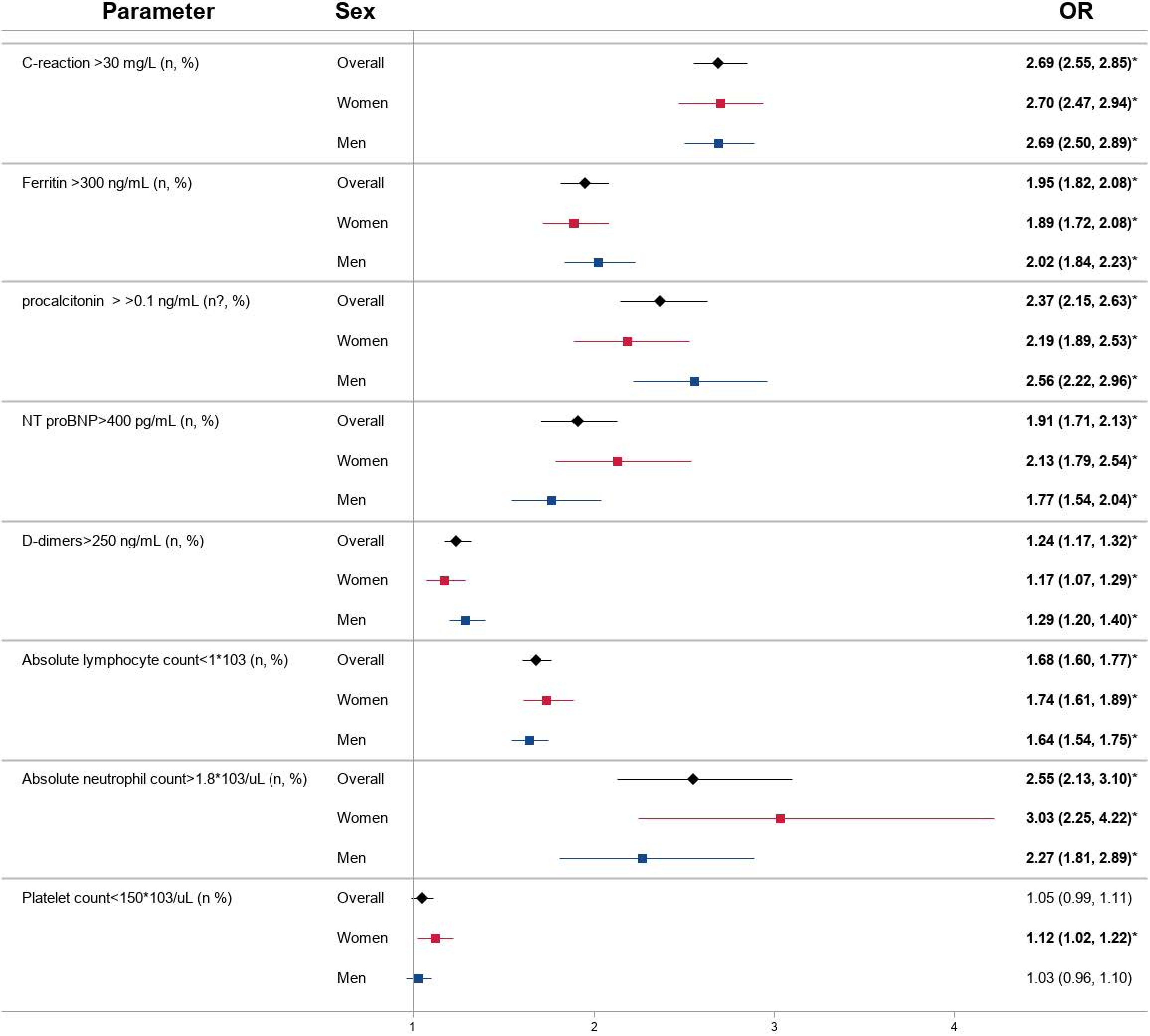

Supplemental Fig 4. The Association between Biomarkers and Hospital Length of Stay > 1 Week in Men and Women with COVID-19

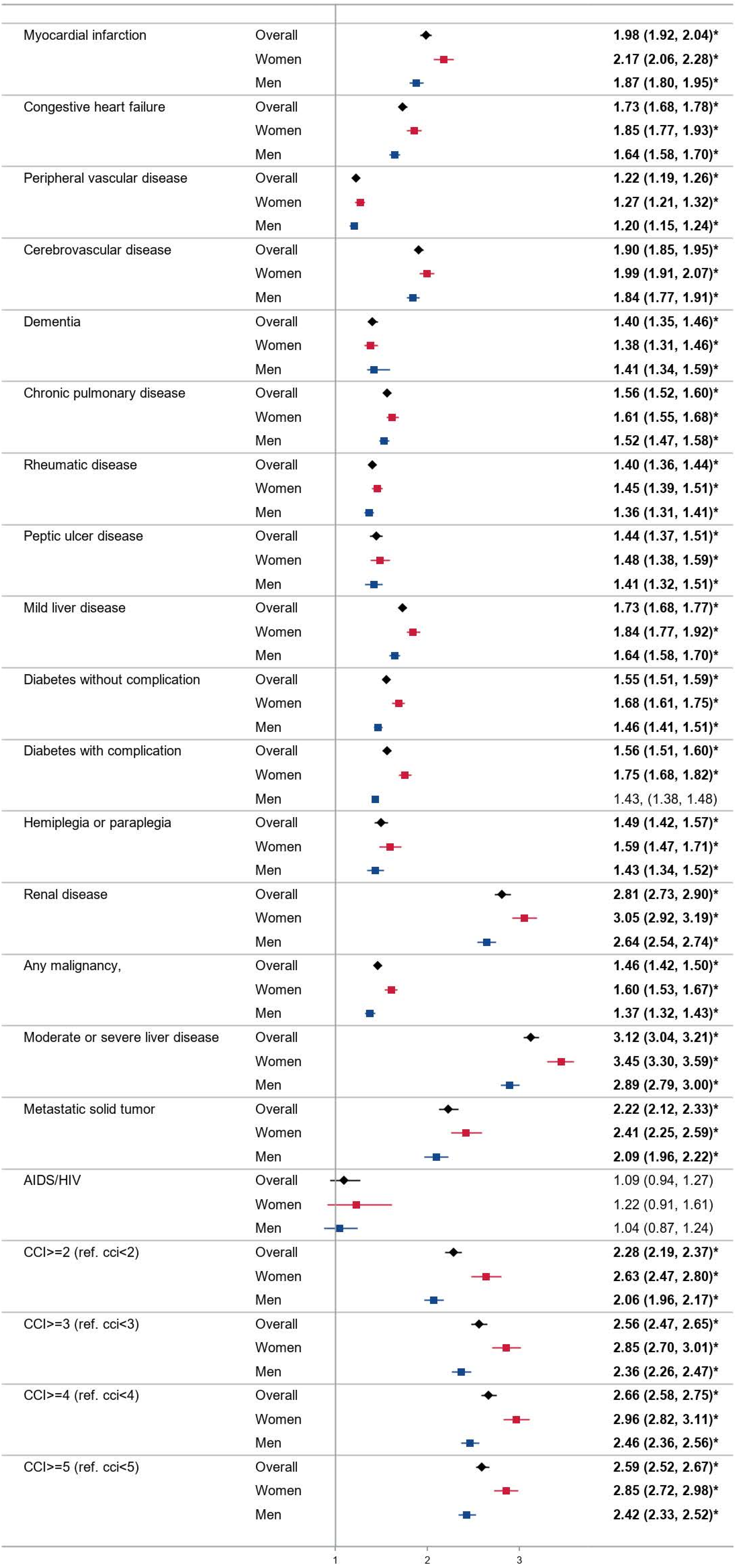

Parameter	Sex	OR
Myocardial infarction	Overall	**1.98 (1.92, 2.04)***
	Women	**2.17 (2.06, 2.28)***
	Men	**1.87 (1.80, 1.95)***
Congestive heart failure	Overall	**1.73 (1.68, 1.78)***
	Women	**1.85 (1.77, 1.93)***
	Men	**1.64 (1.58, 1.70)***
Peripheral vascular disease	Overall	**1.22 (1.19, 1.26)***
	Women	**1.27 (1.21, 1.32)***
	Men	**1.20 (1.15, 1.24)***
Cerebrovascular disease	Overall	**1.90 (1.85, 1.95)***
	Women	**1.99 (1.91, 2.07)***
	Men	**1.84 (1.77, 1.91)***
Dementia	Overall	**1.40 (1.35, 1.46)***
	Women	**1.38 (1.31, 1.46)***
	Men	**1.41 (1.34, 1.59)***
Chronic pulmonary disease	Overall	**1.56 (1.52, 1.60)***
	Women	**1.61 (1.55, 1.68)***
	Men	**1.52 (1.47, 1.58)***
Rheumatic disease	Overall	**1.40 (1.36, 1.44)***
	Women	**1.45 (1.39, 1.51)***
	Men	**1.36 (1.31, 1.41)***
Peptic ulcer disease	Overall	**1.44 (1.37, 1.51)***
	Women	**1.48 (1.38, 1.59)***
	Men	**1.41 (1.32, 1.51)***
Mild liver disease	Overall	**1.73 (1.68, 1.77)***
	Women	**1.84 (1.77, 1.92)***
	Men	**1.64 (1.58, 1.70)***
Diabetes without complication	Overall	**1.55 (1.51, 1.59)***
	Women	**1.68 (1.61, 1.75)***
	Men	**1.46 (1.41, 1.51)***
Diabetes with complication	Overall	**1.56 (1.51, 1.60)***
	Women	**1.75 (1.68, 1.82)***
	Men	1.43, (1.38, 1.48)
Hemiplegia or paraplegia	Overall	**1.49 (1.42, 1.57)***
	Women	**1.59 (1.47, 1.71)***
	Men	**1.43 (1.34, 1.52)***
Renal disease	Overall	**2.81 (2.73, 2.90)***
	Women	**3.05 (2.92, 3.19)***
	Men	**2.64 (2.54, 2.74)***
Any malignancy,	Overall	**1.46 (1.42, 1.50)***
	Women	**1.60 (1.53, 1.67)***
	Men	**1.37 (1.32, 1.43)***
Moderate or severe liver disease	Overall	**3.12 (3.04, 3.21)***
	Women	**3.45 (3.30, 3.59)***
	Men	**2.89 (2.79, 3.00)***
Metastatic solid tumor	Overall	**2.22 (2.12, 2.33)***
	Women	**2.41 (2.25, 2.59)***
	Men	**2.09 (1.96, 2.22)***
AIDS/HIV	Overall	1.09 (0.94, 1.27)
	Women	1.22 (0.91, 1.61)
	Men	1.04 (0.87, 1.24)
CCI>=2 (ref. cci<2)	Overall	**2.28 (2.19, 2.37)***
	Women	**2.63 (2.47, 2.80)***
	Men	**2.06 (1.96, 2.17)***
CCI>=3 (ref. cci<3)	Overall	**2.56 (2.47, 2.65)***
	Women	**2.85 (2.70, 3.01)***
	Men	**2.36 (2.26, 2.47)***
CCI>=4 (ref. cci<4)	Overall	**2.66 (2.58, 2.75)***
	Women	**2.96 (2.82, 3.11)***
	Men	**2.46 (2.36, 2.56)***
CCI>=5 (ref. cci<5)	Overall	**2.59 (2.52, 2.67)***
	Women	**2.85 (2.72, 2.98)***
	Men	**2.42 (2.33, 2.52)***

Supplemental Fig 5. The Association between Comorbidities and Mortality in Hospitalized Men and Women Only (Sensitivity Analysis 1)

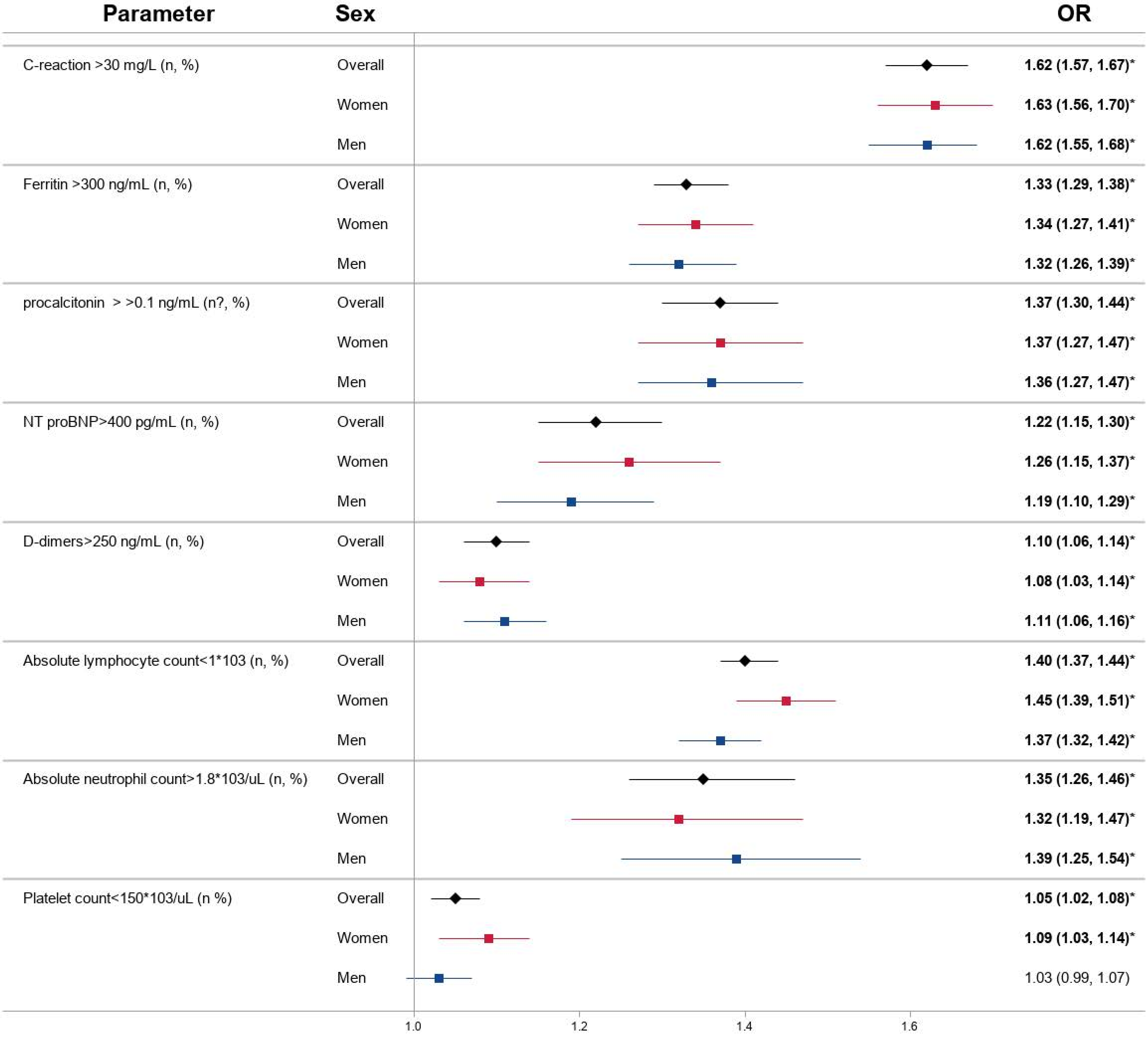

Supplemental Fig 6. The Association between Biomarkers and Mortality in Hospitalized Men and Women Only (Sensitivity Analysis 2)

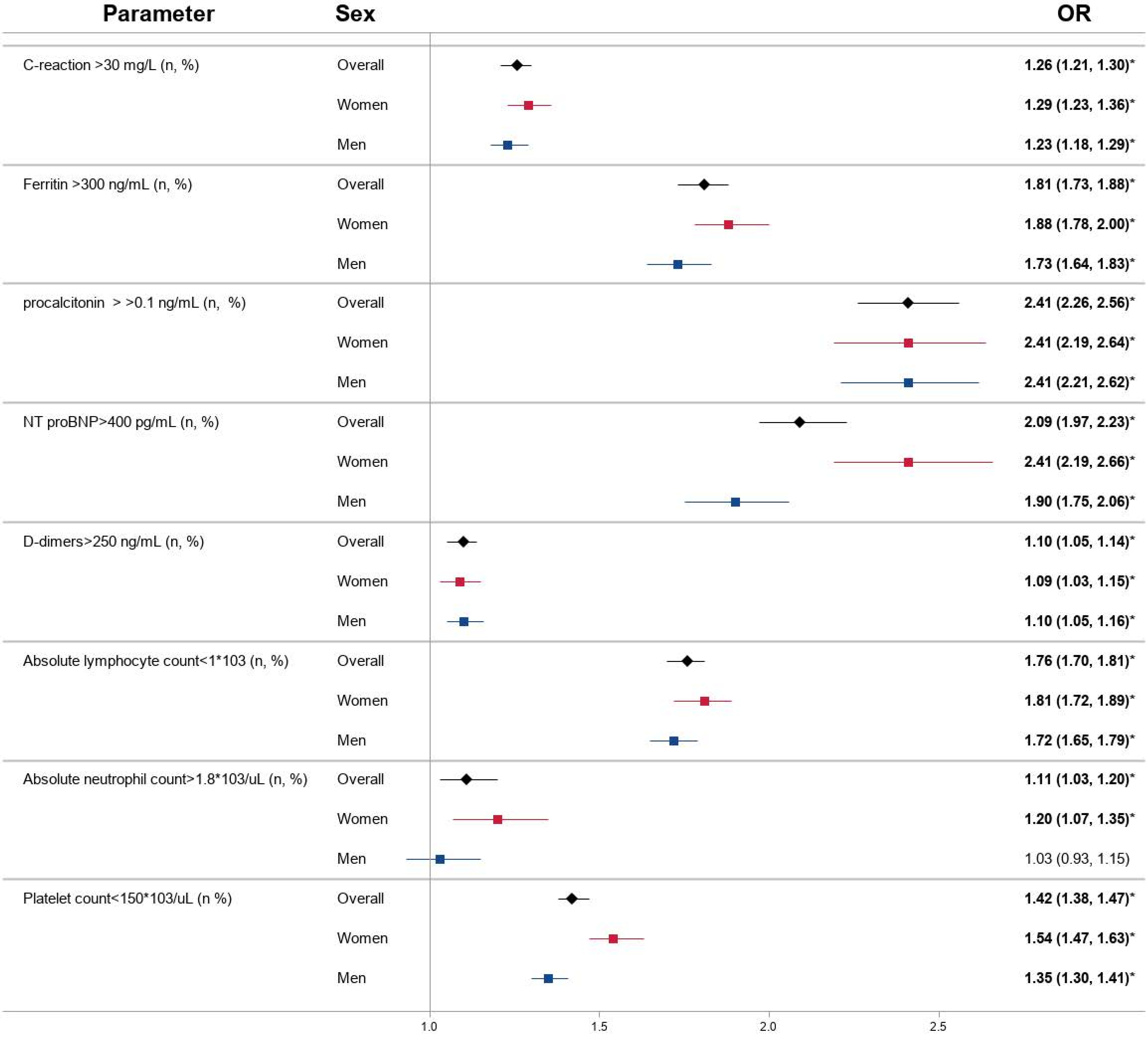

Supplemental Fig 7. The Association between Comorbidities and Mortality in Men and Women with Complete Vitals (Sensitivity Analysis 3)

Parameter	Sex	OR
Myocardial infarction	Overall	1.96 (1.84, 2.08)*
	Women	1.97 (1.78, 2.18)*
	Men	1.96 (1.81, 2.12)*
Congestive heart failure	Overall	1.71 (1.62, 1.81)*
	Women	1.75 (1.61, 1.91)*
	Men	1.69 (1.57, 1.82)*
Peripheral vascular disease	Overall	1.21 (1.15, 1.28)*
	Women	1.26 (1.16, 1.37)*
	Men	1.18 (1.10, 1.27)*
Cerebrovascular disease	Overall	2.18 (2.06, 2.31)*
	Women	2.34 (2.14, 2.56)*
	Men	2.07 (1.92, 2.24)*
Dementia	Overall	1.52 (1.42, 1.64)*
	Women	1.51 (1.35, 1.68)*
	Men	1.54 (1.39, 1.70)*
Chronic pulmonary disease	Overall	1.62 (1.53, 1.72)*
	Women	1.65 (1.51, 1.80)*
	Men	1.61 (1.50, 1.73)*
Rheumatic disease	Overall	1.35 (1.28, 1.43)*
	Women	1.34 (1.23, 1.45)*
	Men	1.36 (1.27, 1.46)*
Peptic ulcer disease	Overall	1.41 (1.29, 1.53)*
	Women	1.35 (1.18, 1.55)*
	Men	1.44 (1.28, 1.62)*
Mild liver disease	Overall	2.08 (1.97, 2.19)*
	Women	2.19 (2.01, 2.38)*
	Men	1.99 (1.85, 2.14)*
Diabetes without complication	Overall	1.54 (1.46, 1.63)*
	Women	1.55 (1.42, 1.69)*
	Men	1.53 (1.42, 1.65)*
Diabetes with complication	Overall	1.56 (1.48, 1.65)*
	Women	1.69 (1.55, 1.84)*
	Men	1.48 (1.38, 1.59)*
Hemiplegia or paraplegia	Overall	1.69 (1.56, 1.84)*
	Women	1.75 (1.53, 1.98)*
	Men	1.65 (1.47,1.84)*
Renal disease	Overall	3.10 (2.92, 3.29)*
	Women	3.13 (2.86, 3.43)*
	Men	3.06 (2.83, 3.32)*
Any malignancy,	Overall	1.52 (1.44, 1.61)*
	Women	1.66 (1.53, 1.80)*
	Men	1.43 (1.33, 1.54)*
Moderate or severe liver disease	Overall	3.35 (3.17, 3.55)*
	Women	3.64 (3.34, 3.98)*
	Men	3.14 (2.91, 3.38)*
Metastatic solid tumor	Overall	2.69 (2.46, 2.93)*
	Women	3.06 (2.68, 3.48)*
	Men	2.44 (2.17, 2.74)*
AIDS/HIV	Overall	0.87 (0.65, 1.14)
	Women	1.03 (0.58, 1.70)
	Men	0.81 (0.58, 1.10)
CCI>=2 (ref. cci<2)	Overall	4.68 (4.11, 5.34)*
	Women	5.14 (4.21, 6.35)*
	Men	4.39 (3.72 ,5.21)*
CCI>=3 (ref. cci<3)	Overall	3.89 (3.55, 4.28)*
	Women	4.06 (3.52, 4.70)*
	Men	3.78 (3.35, 4.29)*
CCI>=4 (ref. cci<4)	Overall	3.50 (3.24, 3.77)*
	Women	3.74 (3.33, 4.20)*
	Men	3.33 (3.02, 3.68)*
CCI>=5 (ref. cci<5)	Overall	3.00 (2.82, 3.24)*
	Women	3.23 (2.93, 3.57)*
	Men	2.84 (2.62, 3.09)*

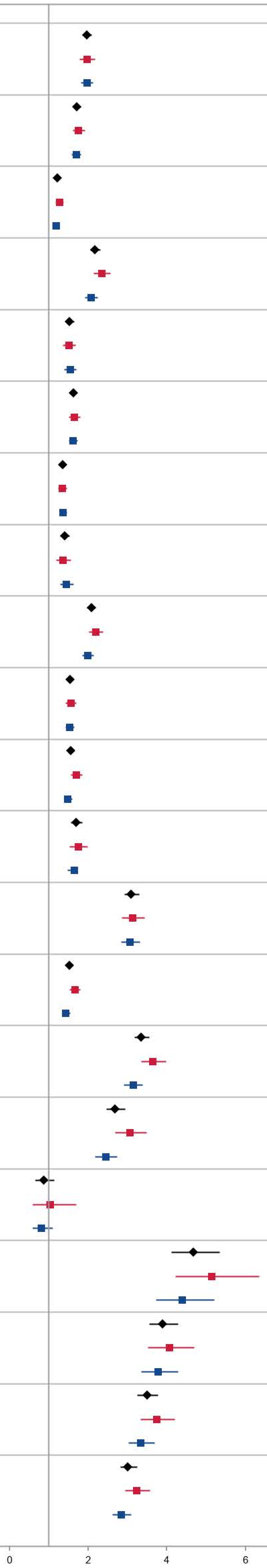

Supplemental Fig 8. The Association between Biomarkers and Mortality in Men and Women with Complete Vitals (Sensitivity Analysis 4)

Parameter	Sex	OR
C-reaction >30 mg/L (n, %)	Overall	**1.27 (1.18, 1.36)***
	Women	**1.28 (1.15, 1.43)***
	Men	**1.26 (1.15, 1.39)***
Ferritin >300 ng/mL (n, %)	Overall	**1.63 (1.50, 1.77)***
	Women	**1.77 (1.58, 2.00)***
	Men	**1.51 (1.35, 1.69)***
procalcitonin > >0.1 ng/mL (n , %)	Overall	**2.02 (1.83, 2.23)***
	Women	**2.08 (1.80, 2.41)***
	Men	**1.97 (1.73, 2.26)***
NT proBNP>400 pg/mL (n, %)	Overall	**2.03 (1.76, 2.36)***
	Women	**2.41 (1.92, 3.04)***
	Men	**1.80 (1.49, 2.19)***
D-dimers>250 ng/mL (n, %)	Overall	0.98 (0.91, 1.06)
	Women	0.96 (0.85, 1.08)
	Men	0.99 (0.89, 1.09)
Absolute lymphocyte count<1*103 (n, %)	Overall	**1.66 (1.57, 1.76)***
	Women	**1.74 (1.60, 1.89)***
	Men	**1.60 (1.48, 1.73)***
Absolute neutrophil count>1.8*103/uL (n, %)	Overall	1.00 (0.88, 1.15)
	Women	0.99 (0.81, 1.21)
	Men	1.01 (0.84, 1.23)
Platelet count<150*103/uL (n %)	Overall	**1.49 (1.40, 1.58)***
	Women	**1.60 (1.44, 1.76)***
	Men	**1.43 (1.32, 1.54)***

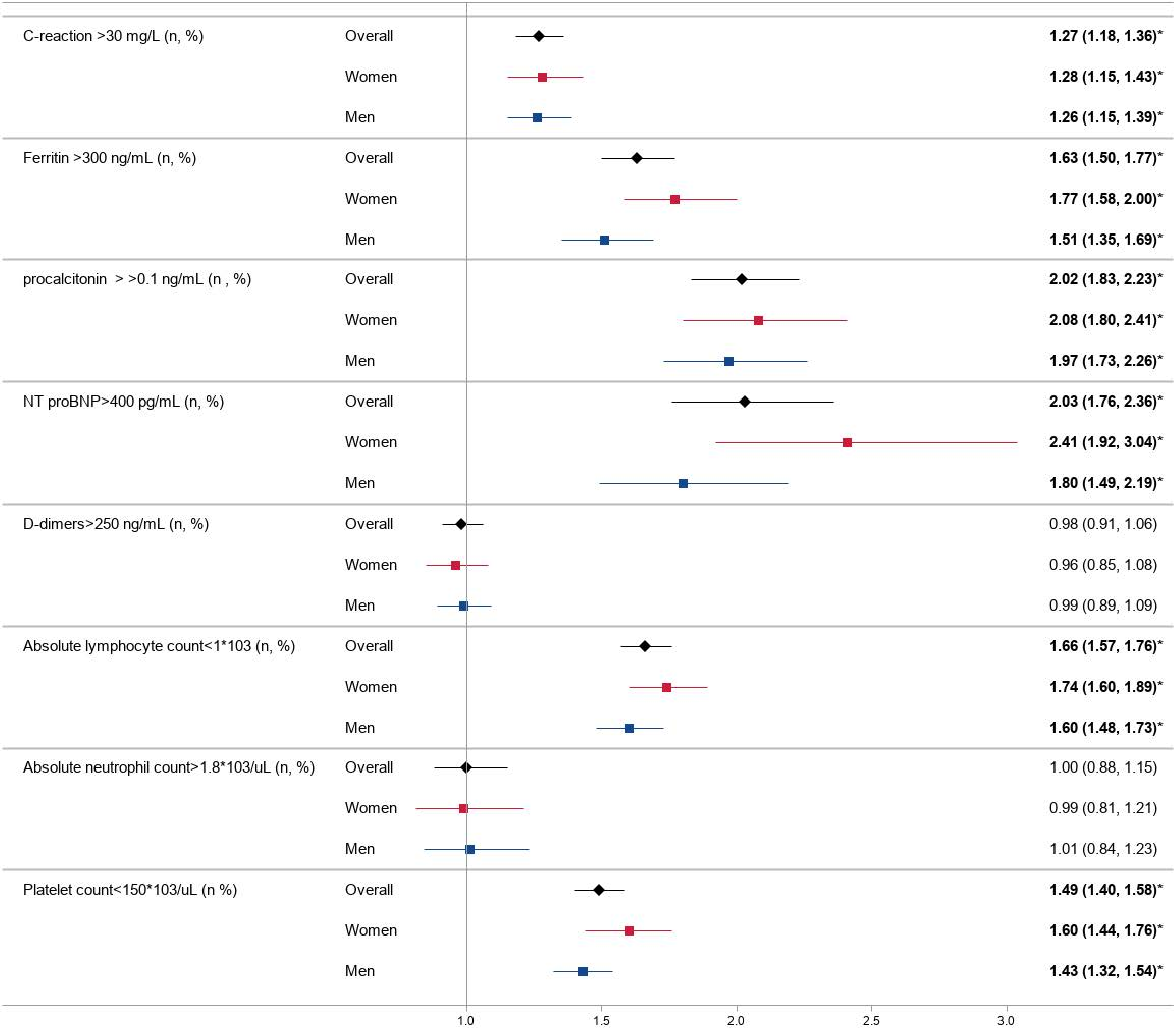

Supplemental Table 1. Key Variable Definitions

**Variable**	**Definition, Codeset**
**Comorbidities (CCI)**	Comorbidities at or before the first admission to hospital or ER due to COVID-19
CCI	2351618
Malignancy	535274723
Congestive heart failure	359043664
Dementia	78746470
Diabetes	719585646
Diabetes with complication	403438288
HIV	73549360
Mild liver disease	494981955
Moderate/severe liver disease	248333963
Metastatic solid tumor	378462283
Myocardial infarction	259495957
Hemiplegia or paraplegia	489555336
Peptic ulcer disease	510748896
Pulmonary disease	514953976
Peripheral vascular disease	376881697
Renal Disease	220495690
Rheumatic arthritis	7650044049
Cerebrovascular disease	652711186
**Biomarkers**	Averaged measurements taken within 15 days prior or after the first admission for COVID-19
C-reactive protein	371622342
Ferritin	317388455
Procalcitonin	610397248
NT proBNP	561166072
D-dimer	475972797
Absolute lymphocyte counts	627523060
Absolute neutrophil count	881397271
Platelet count	167697906
Troponin	623367088
